# Predicting and Managing Hepatocellular Carcinoma Recurrence After Liver Transplant: A Single-Center Experience 2012–2024

**DOI:** 10.3390/cancers18050721

**Published:** 2026-02-24

**Authors:** Jesse Civan, Madison Force, Ali Raza Shaikh, Adam Bodzin, Daniel Lin

**Affiliations:** 1 Division of Gastroenterology and Hepatology, Department of Medicine, Thomas Jefferson University, Philadelphia, PA 19107, USA; madison.force@jefferson.edu; 2 Department of Medical Oncology, Thomas Jefferson University, Philadelphia, PA 19107, USA; aliraza.shaikh@jefferson.edu (A.R.S.); daniel.lin@jefferson.edu (D.L.); 3 Department of Surgery, Thomas Jefferson University, Philadelphia, PA 19107, USA; adam.bodzin@jefferson.edu

**Keywords:** HCC, hepatocellular carcinoma, liver transplantation, immunotherapy, HCC recurrence, RETREAT score

## Abstract

**Background**: Hepatocellular carcinoma (HCC) is a major cause of mortality in the United States, but it can be cured with orthotopic liver transplant (OLT) in selected patients. Despite curative intent, post-OLT recurrence can occur in up to 15% of patients. The need for a program of post-OLT surveillance is widely accepted but the specifics of an optimal program have not been established. There is interest in identifying lower-risk cohorts of patients in whom an abbreviated strategy of surveillance may prove adequate, utilizing tools such as the RETREAT score. Unique challenges are posed in the post-transplant population regarding safety and tolerability of systemic therapy for HCC recurrence, suggesting early detection is beneficial. **Methods**: This was a single-center retrospective analysis of characteristics and outcomes for all patients undergoing transplant at our center between 1 January 2012 and 31 December 2024. Diagnosis of HCC was determined by histological confirmation or Liver Imaging and Reporting Data System (LI-RADS) 5 findings on contrast-enhanced cross-sectional imaging. RETREAT scores were calculated for all patients. **Results**: During the study period, 923 transplants were performed, of which 329 (35.6%) were for HCC. Post-OLT recurrence occurred in 36 (10.9%) of these. Recurrence was associated with the presence of any viable tumor on explant surgical pathology, the presence of a viable tumor beyond Milan Criteria, the presence of microvascular invasion, a larger diameter of viable tumor on explant, and a higher RETREAT score. Although higher RETREAT scores were associated with post-OLT recurrence, one-third of patients who experienced post-OLT recurrence had RETREAT scores of 0 or 1. RETREAT scores did not correlate with the time interval between transplant and HCC recurrence. Systemic therapy proved challenging, with 10/25 patients receiving systemic therapy for post-OLT recurrence having to stop or alter regimens due to the severity of adverse effects. **Conclusions**: The rates of post-transplant recurrence and the experience of patients managed with systemic therapy for post-OLT recurrence in our experience were in line with previously published data. Due to the overall low RETREAT scores, the sensitivity of the RETREAT score in identifying patients at risk for post-OLT recurrence was limited, and the low RETREAT score had very limited incremental negative predictive value for identifying a low-risk population. This suggested that a broad screening strategy for post-OLT recurrence may be better than a personalized strategy in which patients with low RETREAT scores receive abbreviated surveillance.

## Simple Summary

Liver transplant can cure hepatocellular carcinoma (HCC), but recurrence can be expected in 10–15% of cases. There is interest in identifying subgroups of patients with particularly low risk for post-transplant recurrence in whom an abbreviated protocol of surveillance for post-transplant HCC recurrence may be acceptable, for example, based on RETREAT scores. We analyzed the outcomes of 923 patients undergoing liver transplant at our center between 2012 and 2024, of which 329 were for HCC. Our observed recurrence rate was 11%. We found that the RETREAT score had limited sensitivity in predicting HCC recurrence, with one-third of patients with post-transplant HCC recurrence having RETREAT scores of 0 or 1. Due to unique challenges in the post-transplant population regarding the provision of systemic therapy for recurrent HCC, early detection is a priority. Our findings suggest that a broad surveillance strategy may be justified.

## 1. Background

Hepatocellular carcinoma (HCC) remains a major cause of morbidity and mortality in the United States. According to estimates from the American Cancer Society, in 2025 there were over 42,000 new cases of primary liver cancer in the United States and over 30,000 deaths attributed to primary liver cancer, making this the 5th leading cause of cancer death in men and the 7th leading cause of cancer death in women [1]. Based on data from the National Cancer Institute Surveillance, Epidemiology, and End Results (SEER) program, the incidence of HCC in the United States is estimated at 6.3 cases per 100,000 person-years [2], although incidence appears to have recently peaked and rates may be decreasing [3].

For selected patients, orthotopic liver transplant (OLT) may prove curative for HCC. However, despite this, recurrence may occur. A 2018 systematic review and meta-analysis of 15 studies reported an overall rate of recurrence post-OLT HCC of 14% [4], with another review reporting rates of post-OLT recurrence ranging from 10% to 16% [5]. HCC remains an important indication for OLT, with the most recently available data from the Scientific Registry for Transplant Recipients (SRTR) demonstrating that HCC was the primary indication for liver transplant for 10.4% of all patients listed for transplant across the United States in 2023, which has remained a stable proportion of patients being listed for this indication over the preceding decade [6].

There is general agreement that some program of surveillance for post-OLT HCC recurrence involving cross-sectional contrast-enhanced imaging is indicated. However, there is at present no consensus regarding the specifics of such a program, including imaging modality, frequency of imaging, and duration of the necessary surveillance period. The current AASLD guideline on HCC published in 2023 advises surveillance for the detection of post-transplant HCC recurrence using multiphasic contrast-enhanced abdominal CT or MRI and chest CT scan, but notes that the optimal timing and duration of post-transplant surveillance is uncertain [7]. The current 2025 European Association for the Study of the Liver (EASL) clinical practice guideline on HCC makes no specific recommendations for post-OLT HCC surveillance [8]. A 2025 guidance statement generated by an expert panel on behalf of the American College of Radiology recommends the use of either MRI or multiphasic contrast-enhanced CT as the modality of choice for this purpose without recommending a schedule of imaging [9]. Current National Comprehensive Cancer Center Network (NCCN) guidelines recommend the same program of surveillance for patients undergoing curative intervention with either liver transplantation or with resection, despite significantly different risks of post-operative recurrence with these interventions, outlining a program of imaging every 3–6 months for the first two years, and then every 6 months thereafter [NCCN Guidelines Version 2.2025 Hepatocellular Carcinoma]. In the absence of a universally accepted program of radiographic surveillance, clinical practice patterns across the US vary in terms of strategies for identifying post-OLT HCC recurrence [5].

The actual costs associated with post-transplant surveillance for HCC recurrence are difficult to assess both in absolute dollar amounts and as a percentage of overall costs associated with liver transplantation for HCC, in part due to the lack of transparency of actual costs in the United States and the complexity of care delivery and payment systems. For these reasons, it is unclear how well published data on these types of costs from other parts of the world utilizing other models of healthcare delivery and payment can be generalized to the United States. Regardless of the absolute costs, investigators have attempted to identify subsets of patients at relatively lower risk for post-transplant HCC recurrence in whom a more abbreviated form of post-OLT HCC surveillance may be adequate and associated with lower costs. Examples of reported predictors of risk of post-transplant recurrence include a viable tumor on explant surgical pathology falling within the Milan Criteria [10], the RETREAT score [11], pre-transplant laboratory parameters including cancer biomarkers such as AFP-L3 and DCP [12], and the neutrophil-to-lymphocyte ratio [13]. Just as there is no universally established protocol for surveillance for post-OLT HCC recurrence in general, there is no clear definition of what an “abbreviated” protocol would entail for a low-risk sub-population. However, proposals for lower intensity surveillance have included going so far as to perform no surveillance at all for patients with RETREAT scores of 0 [11].

The management of post-OLT recurrence of HCC poses unique challenges. If recurrence is localized and confined to the liver, locoregional therapies such as resection, transarterial chemoembolization, transarterial radioembolization, or microwave or radiofrequency ablation may be utilized [14]. On the other hand, if recurrence occurs beyond the limitations of locoregional therapies, systemic therapy is recommended. As a consequence of the immunosuppression therapy necessary after liver transplant to protect the allograft from rejection, systemic treatment options are more limited, and particular attention needs to be paid to toxicity management. Although immunotherapy combinations are utilized in the first-line setting for patients with advanced, metastatic disease in the general population, these therapies may counteract immunosuppression medications and pose a significant risk of rejection of the liver allograft, graft loss, and death in the post-transplant population. This has led to recommendations against the use of immunotherapy in the post-transplant setting in both current AASLD and EASL guidelines [7,8].

Data pertaining to the safety concerns of immunotherapy post-transplant arise from multiple case reports and institutional case series, which demonstrate variable rates of graft rejection [15,16,17]. Two pooled analyses have reported a rate of graft rejection of between 20 and 28% [18,19]. A systematic review by Ziogas et al. noted a 40% rate of graft rejection in a cohort of 15 patients receiving immunotherapy post LT [20]. In addition, a global study reported that out of 386 HCC patients receiving immunotherapy in the OLT setting, 22.1% experienced graft rejection with immunotherapy [21]. Data also remains limited on whether immunosuppressing medications may blunt the anti-tumor response conferred by immune checkpoint inhibitors [22]. Therefore, typical systemic treatment options post-OLT include vascular endothelial growth factor receptor (VEGF)-directed tyrosine kinase inhibitors (TKIs). Such agents include lenvatinib or sorafenib in the first line of therapy and cabozantinib or regorafenib in later lines. Because liver transplant recipients have generally been excluded from most systemic therapy clinical trials, data regarding safety and tolerability of TKIs remains limited to retrospective studies and analyses.

Thus, given more limited systemic treatment options post-OLT, it is often reasonable to approach oligometastatic HCC recurrence with locoregional therapy first and delay initiation of systemic therapy for either greater burden of disease or more rapid progression. As a consequence of these unique challenges to management of post-transplant HCC recurrence with systemic therapies, there is likely clinical benefit to early diagnosis of post-transplant HCC recurrence when it does occur. Likewise, this line of reasoning implies that if criteria are identified to designate a sub-population of patients undergoing OLT for HCC as sufficiently low risk for post-OLT recurrence so as to merit an abbreviated program of surveillance, then these criteria should be quite rigorous and carry a very high negative predictive value for post-OLT recurrence.

In this study, we aimed to describe our single-center experience with post-transplant HCC recurrence and, in particular, explore the performance of the RETREAT score in identifying a population of patients at lower risk for post-OLT recurrence in whom an abbreviated program of radiographic surveillance might be reasonable after OLT.

## 2. Methods

This was a single-center retrospective study based on a chart review of patients treated at Thomas Jefferson University Hospital. All cases of liver transplants performed between 1 January 2012 and 31 December 2024 were included. The study period was chosen due to limitations in the accessibility of older records in our center’s current electronic medical record. Patient charts were manually reviewed to abstract relevant clinical data, including demographics, laboratory data, radiographic data, histological data, clinical interventions, and clinical outcomes.

Patients were considered to have undergone OLT for HCC if they had either a known diagnosis of HCC prior to the transplant or had incidentally identified HCC on explant surgical pathology. The pre-transplant HCC diagnosis was based on either a definite radiographic diagnosis with Liver Imaging Reporting and Data System (LI-RADS) 5 findings on either a contrast-enhanced MRI or a triple-phase CT, or on a histological diagnosis determined prior to the transplant.

LI-RADS classifications were determined prospectively using the most current version of the LI-RADS algorithm in clinical practice at that time. Our study period encompassed several updates of the LI-RADS algorithm, from the original 2011 version to the current 2024 version [23]. Pre-transplant radiographic staging of HCC was determined based on the number and size of definite HCC lesions, counting LI-RADS 5 lesions, LI-RADS TR-viable lesions, or enhancing/viable lesions previously determined on biopsy to represent HCC. Lesions that were not counted toward radiographic staging included LI-RADS 4, LI-RADS 3, LI-RADS TR-equivocal, and LI-RADS TR-non-progressing lesions.

Surveillance for post-transplant HCC recurrence was determined by our center’s standard clinical protocol during the study period, which included an abdominal MRI every 3 months for the first year after transplant, an abdominal MRI every 6 months in the second year after transplant, and an annual abdominal MRI for years 3–5 after transplant. At that time, our standard protocol did not mandate a chest CT. AFP measurements were checked per protocol along with MRI exams.

Recurrence was identified by either histologic or definite radiographic evidence (LI-RADS 5 findings on contrast-enhanced MRI or triple-phase CT) within 5 years of transplant. HCC more than 5 years out from transplant was not considered recurrence, but rather de novo HCC.

Statistical analysis was performed with IBM SPSS Version 29.0.0.0, with the exception of Microsoft Excel Version 2601 (Build 19628.20204) being used to calculate 95% confidence intervals for positive and negative predictive values. The variables selected for univariate analysis were selected based on clinical relevance and as established in the literature. Variables included in multivariate analysis were those identified as statistically significant in univariate analysis. Categorical variables were assessed with the chi-square test. Continuous variables were assessed using an independent samples *t*-test. The 95% confidence intervals for positive and negative predictive values were calculated using the Wilson Score method. Patient survival was assessed by Kaplan–Meier analysis. Multivariate analysis was conducted with Cox proportional hazards regression, with time calculated from the date of the liver transplant to either the date of recurrence of HCC or the date of last clinical follow-up.

## 3. Results

None of the patients undergoing liver transplant for HCC at our center had received immunotherapy prior to transplant, although a small number did experience pre-transplant systemic therapy with sorafenib.

**Figure 1:**
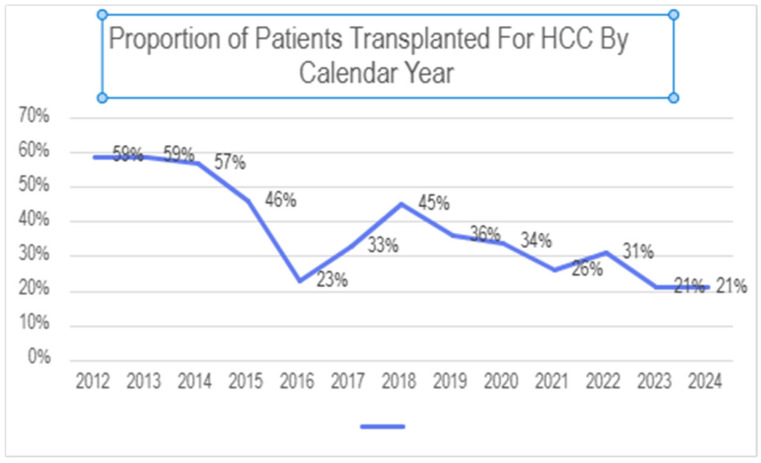
Proportion of patients undergoing liver transplant at our center for HCC.

During the study period, a total of 923 liver transplants were performed at our institution. Of these, 329 (35.6%) were performed for HCC. The proportion of patients undergoing transplant for HCC varied significantly by year, ranging from a peak of 59% of transplants early in this period to a nadir of 21% most recently (see Figure 1).

Baseline patient characteristics for the entire population, along with comparisons between the sub-population with and without HCC, are summarized in Table 1 below. The mean and median age at transplant were 58.6 and 60.3 years, respectively. The HCC group was older than the non-HCC group, with a mean age of 62.9 versus 56.3 (*p* < 0.01) and a median age of 63.5 versus 57.6, respectively. The overall population was predominantly male, with 613 (66.4%) males and 310 (33.6%) females. Comparing the HCC to the non-HCC groups, the HCC group was more male predominant (80%) than the non-HCC group (59%) (*p* < 0.001). The overall population was predominantly White, with 77.5% of the population being White, 12% Black, 5.1% Hispanic, 4% Asian and 1.4% “other”. There was no statistically significant difference in the composition of the HCC and non-HCC groups based on race. In the overall population, the etiology of underlying chronic liver disease was viral in 28.4%, alcohol-related in 28.6%, MASH in 14.5%, and a combination of these risk factors or “other” in 28.5%. The HCC group encompassed more patients with viral hepatitis and fewer patients with alcohol induced liver disease or with multiple/“other” etiologies (*p* < 0.001). For the overall population, the mean and median wait list times were 7.0 and 4.5 months, respectively. Patients in the HCC group had longer wait times in comparison to the non-HCC group, with mean wait times of 8.6 versus 6.1 months (*p* < 0.001) and median wait times of 8.1 versus 1.6 months, respectively. For the overall population, the mean and median clinical follow-up were 66.1 and 60.4 months, respectively. The HCC group had a longer clinical follow-up compared to the non-HCC group, with a mean of 78.5 versus 59.2 months (*p* < 0.001) and a median of 75.6 versus 53.3 months, respectively.

Among the HCC subgroup of patients, 36 (10.9%) experienced documented post-OLT recurrence of HCC within 5 years of transplant. One additional patient was observed to have HCC recurrence a little over 7 years after transplant. Based on our a priori definition of “recurrence” being within 5 years, this individual was not counted among the 36 patients with post-OLT HCC recurrence, and rather was considered to have developed de novo HCC after transplant.

The baseline characteristics of the patients transplanted for HCC, along with a comparison of baseline characteristics between the subgroups with and without post-transplant recurrence, are summarized in Table 2 below. There was a trend toward the group of HCC subjects with recurrence being older than those without recurrence (mean age of 64.3 versus 62.7 years and median age 66.1 versus 63.2 years); however, this was not a statistically significant difference. Likewise, there was a trend toward the group of HCC patients with recurrence being more male predominant than in the HCC group without recurrence (86.1% male versus 79.5% male); however, this was not a statistically significant difference. The distribution of race between the group of subjects with HCC recurrence showed a trend toward a higher proportion of White and a lower proportion of Black patients; however, this was not a statistically significant difference. There was a trend toward the underlying etiology of chronic liver disease being more likely to be alcohol or MASH in the group with HCC recurrence, and more likely to be viral in the group with HCC without recurrence; however, this was not a statistically significant difference.

In terms of pre-transplant HCC treatment, the majority of HCC patients (88.4%) underwent locoregional therapy either as bridging or downstaging therapy prior to transplant. Reasons for not performing locoregional therapy prior to transplant included patient not having a definite HCC diagnosis prior to transplant (with the condition either unsuspected and evident only on explant, or suspected but confirmed only on explant), patient being deemed too clinically decompensated to tolerate locoregional therapy prior to transplant, patient being managed with a “wait to ablate” strategy at the time of transplant, and patient undergoing liver transplant prior to scheduled locoregional therapy. There was a trend toward a higher proportion of HCC patients with recurrence having undergone pre-transplant locoregional therapy compared to those with HCC without recurrence (97.2% versus 87.4%); however, this difference did not meet statistical significance. There was a trend toward a larger proportion of patients with HCC with recurrence having undergone downstaging to transplant compared to the group with HCC without recurrence (16.7% versus 10.2%); however, this difference did not meet statistical significance. None of the patients in either group underwent immunotherapy prior to transplant, as this was not our institutional clinical practice during the study period.

In terms of explant findings, the majority (62.9%) of patients with HCC had some viable HCC tumor identified on explant surgical pathology. A larger proportion of patients with HCC with recurrence had some viable tumor identified compared to the patients with HCC without recurrence (86.1% versus 60.1%) (*p* < 0.02). Of the overall patients with HCC, 12.2% had a viable HCC tumor beyond the Milan Criteria apparent on explant surgical pathology. The patients with HCC with recurrence were more likely than the patients with HCC without recurrence to have had viable HCC beyond the Milan Criteria apparent on explant surgical pathology (36.1% versus 9.2%) (*p* < 0.001). Of the 40 subjects with viable HCC identified on explant surgical pathology, 32.5% had post-transplant recurrence and 67.5% did not. Microvascular invasion was identified in 17 (5.2%) patients in the HCC group. The patients with HCC with recurrence were more likely to have had microvascular invasion identified on explant surgical pathology than the patients with HCC without recurrence (19.4% versus 3.4%) (*p* < 0.001). In the overall group, the mean diameter of the large focus of viable HCC on the explant was 1.1 cm, and the median was 0.6 cm. Patients with HCC with recurrence had larger diameters of the largest focus of viable HCC on explant compared to patients with HCC without recurrence, with a mean of 2.5 cm versus 0.9 cm (*p* < 0.001) and a median of 2 cm versus 0.5 cm. Results of univariate analysis of predictors of recurrence are summarized above in Table 2 and multivariate analysis below in Table 3. 

Regarding the most recent AFP measurement leading up to the time of transplant, for the overall population of patients transplanted with HCC, the mean was 34, the median was 5, and the range was from 1 to 2206. Comparing the group of HCC patients with recurrence to those without recurrence, those with recurrence had a trend toward high AFP measurements, with a mean of 42.5 versus 33.4 and a median of 8.5 versus 5, but this difference did not meet statistical significance.

**Figure 2:**
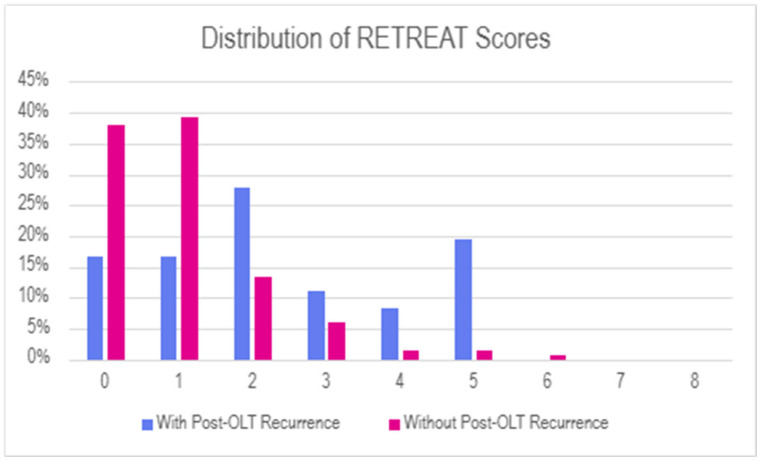
Histogram of RETREAT scores in the population of patients undergoing OLT for HCC, comparing subgroups of patients with POST-OLT recurrence versus those without.

RETREAT scores were higher in the group with recurrence, with a mean RETREAT score of 2.4 and a median RETREAT score of 2.0 in the group with recurrence versus a mean RETREAT score of 1.0 and a median RETREAT score of 1.1 in the group without recurrence. Of note, almost all subjects in both groups had RETREAT scores of no more than 5. Two subjects, both in the group without recurrence, had a RETREAT score of 6. None of the patients in either group had RETREAT scores greater than 6. Distribution of RETREAT scores is summarized in Figure 2. 

**Figure 3:**
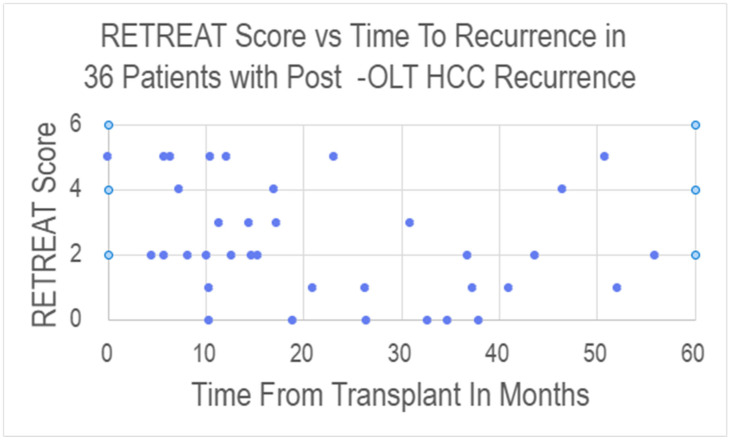
Scatter plot showing the relationship between RETREAT score and time from transplant to documentation of recurrence for the 36 patients who experienced post-OLT HCC recurrence.

For the 36 patients with post-OLT recurrence, the time from transplant to recurrence ranged from 0 to 55.9 months, with a mean time from OLT to recurrence of 22.5 months and a median of 17.1 months. There was no statistically significant correlation between RETREAT scores and time to recurrence (see Figure 3 and Table 4).

**Figure 4:**
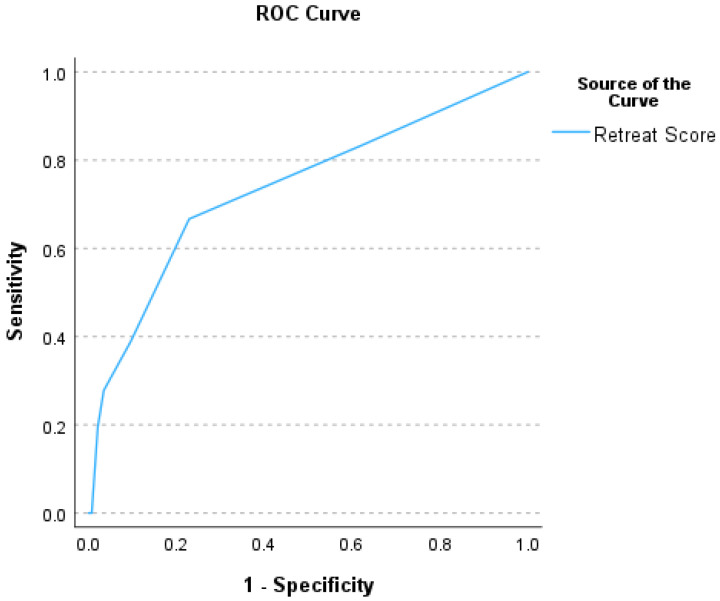
Receiver operator characteristic (ROC) curve summarizing the performance of the RETREAT scores in predicting post-OLT HCC recurrence (area under the ROC curve of 0.74).

In terms of identifying a lower-risk patient population by RETREAT score, we assessed the diagnostic performance of the RETREAT score using all possible cutoff values (see Table 5 and ROC curve in Figure 4). 

**Figure 5:**
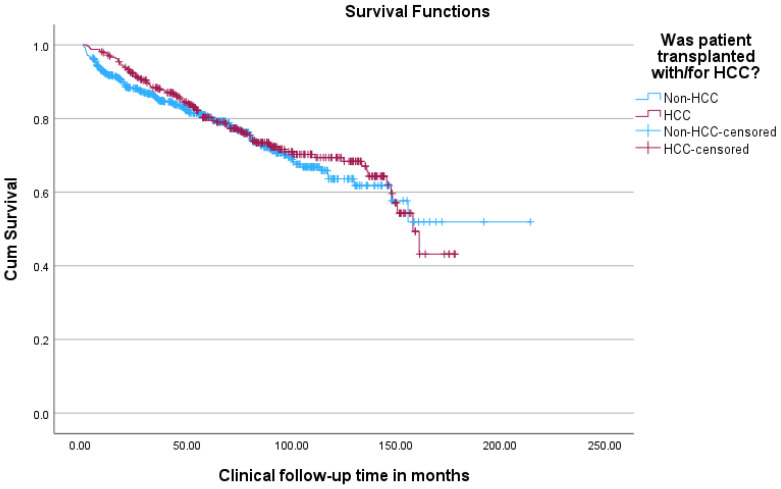
Kaplan–Meier survival curves comparing overall survival of patients transplanted with versus without HCC.

In terms of overall survival, patients transplanted with HCC had better outcomes than patients transplanted without HCC, with a 1, 3, 5, and 10-year survival of 97%, 88%, 80%, and 69%, respectively, for the HCC group versus a 1, 3, 5, and 10-year survival of 92%, 85%, 80%, and 64%, respectively, for the non-HCC group (see Table 6 and Figure 5).

**Figure 6:**
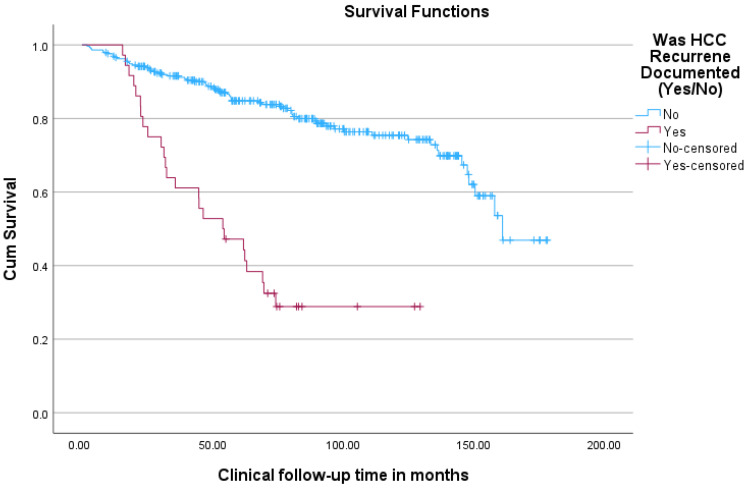
Kaplan–Meier curves comparing overall survival (from time of transplant) for HCC patients undergoing transplant between groups of patients with and without post-transplant HCC recurrence.

Within the group of patients transplanted with HCC, survival was superior for patients without post-OLT recurrence in comparison to those with post-OLT recurrence (see Figure 6).

**Figure 7:**
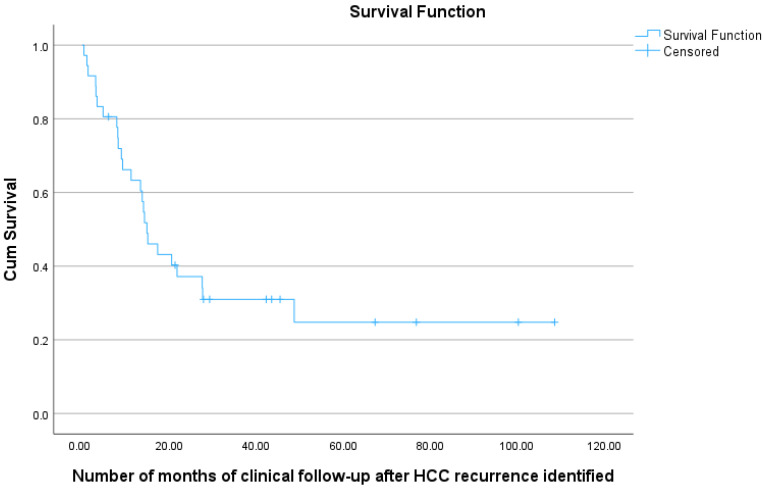
Kaplan–Meier curve showing overall survival (from time of identification of post-OLT HCC recurrence) for 36 HCC patients undergoing transplant, with documented post-transplant HCC recurrence.

For the 36 patients who experienced recurrence, the 1-year survival from the time of HCC recurrence was 63%, the 2-year survival was 37%, and the 3-year survival was 31% (see Figure 7 and Table 7).

For the patients with post-OLT HCC recurrence, the initial site of recurrence was noted as nodal or masses in the abdominopelvic region in about 25.0% of patients, followed by 22.2% in the liver, 16.7% in the lungs, and 11.1% in the bones (see Table 8). Additionally, 25% of patients had multiple sites of synchronous recurrence.

In the 36 patients with recurrence, 6 were managed with locoregional treatments alone and did not require or opted not to pursue systemic treatments. Six patients were noted to have a significantly poor Eastern Cooperative Oncology Group (ECOG) performance status score of 2–3 and clinical decline, and therefore no systemic treatment was recommended or pursued. Of those patients who received systemic therapy, 24 received first-line treatment, 13 received second-line treatment, and 5 received third-line treatment. Among those receiving first-line treatment, 13 patients received lenvatinib and 10 patients received sorafenib, with only 1 patient receiving cytotoxic chemotherapy. The most common second-line treatment was cabozantinib (7 patients), while the other TKIs administered included sorafenib and regorafenib. Of note, there were 2 patients who enrolled in a clinical trial. There was 1 patient who received post-transplant immunotherapy with atezolizumab plus bevacizumab after progression on frontline Lenvatinib (See Table 9).

Of those who received systemic treatment, discontinuation of therapy was noted in 13 patients due to treatment-related toxicities across all lines of therapy. Grading of adverse events was difficult to ascertain given the variability in chart notes and documentation among different providers; however, there were multiple symptom-related toxicities observed. Fatigue was common among patients who received TKI therapy, which was noted in 7 patients who received lenvatinib, 4 patients who received sorafenib, 3 patients who received cabozantinib, and 1 patient who received regorafenib. Loss of appetite and weight loss were observed in 4 patients on cabozantinib, 3 patients on sorafenib, and 2 patients on lenvatinib. Diarrhea was reported in 3 patients on cabozantinib, 3 patients on sorafenib, and 2 patients on lenvatinib. Hand-foot syndrome was noted in 2 patients on sorafenib, 1 patient on cabozantinib, and 1 patient on lenvatinib. Of note, Grade 3 proteinuria, documented by urine protein/creatinine ratio and 24 h urine protein collection, was reported in 2 patients who received lenvatinib, necessitating discontinuation. For those receiving cytotoxic chemotherapy, neuropathy was observed in one patient who received oxaliplatin, and interstitial pneumonitis was seen in another patient, who required treatment with steroids. In the 1 patient who received atezolizumab plus bevacizumab, treatment was ultimately discontinued after two cycles due to immune-related hepatitis, which was treated with steroids and additional immunosuppression. Importantly, a non-targeted liver biopsy was performed for this patient, which confirmed no evidence of graft rejection. See Supplemental Table S5 for further details.

Among those patients who received first-line systemic therapy, the median and mean duration of first-line treatment prior to progression, toxicity or death was 4 months and 6 months, respectively. We excluded one patient who received sorafenib in the post-operative setting for 2 months in this efficacy assessment. One patient notably received lenvatinib for 30 months prior to progression, and another currently remains on lenvatinib at the time of this data analysis. There were variable TKI dosing strategies observed, which differed among clinicians; these included weekly uptitration to the maximum tolerated dose, as well as starting at the maximum recommended dose and reactive dose modification due to toxicity.

Furthermore, the most common immunosuppression among patients who received systemic therapy was tacrolimus (15 patients). Six patients received sirolimus, 1 patient received cyclosporine, 1 patient received a combination of tacrolimus and mycophenolate mofetil. See Supplemental Table S5.

## 4. Discussion

The medical literature describes strategies to identify patients at relatively low risk for post-transplant HCC recurrence in whom an abbreviated protocol of radiographic surveillance for HCC recurrence might be adequate and reduce costs. Although it is appealing to reduce both real dollar costs, as well as non-tangible costs (time and stress of patients, time and energy of liver transplant coordinators, risks of false-positive findings on surveillance imaging), the costs specific to a post-transplant radiographic surveillance program to monitor for HCC recurrence likely represent a small proportion of the overall care of these patients. Furthermore, transplant programs are deeply invested in these patients, wanting the patients to derive as much benefit as possible from their liver allograft not only for the sake of the individual patient but also from the standpoint of stewardship of the precious societal resource of cadaveric donors or of justifying the risk to donors in cases of living donor liver transplant. Additionally, due to unique challenges in the management of post-transplant HCC recurrence with systemic therapies, patients would likely benefit from early detection with a robust program of post-transplant surveillance if this allows for early detection at a stage more likely to respond to locoregional therapy without requiring the initiation of systemic therapy at the time of post-transplant diagnosis of recurrence. Therefore, a very high negative predictive value of a scoring system to identify patients at low risk would be needed to justify the adoption of this strategy for the purpose of cost containment.

In our center’s experience, just over one-third of patients who underwent liver transplant from 2012 to 2024 had HCC. The great majority of these had a known diagnosis of HCC prior to transplant, and a small fraction were diagnosed incidentally on explant surgical pathology. The great majority—mostly patients presenting within the Milan Criteria undergoing loco-regional therapy as a bridge to transplant—had undergone locoregional therapy prior to transplant, but around 11% underwent downstaging, having initially presented beyond the Milan Criteria. A small number of patients had experienced systemic therapy with tyrosine kinase inhibitors, and none had pre-transplant immunotherapy. Using our center’s clinical protocol of surveillance (at that time an abdominal MRI every 3 months for one year, then every 6 months for one year, then once annually for the following 3 years, with AFP measurements but not mandating CT chest), our recurrence rate was 10.9% overall.

In our data, RETREAT scores were generally low, with only two patients having a RETREAT score >5 and both of these patients in the subgroup of patients who did not experience recurrence. Of the patients who did experience recurrence, about one-third had a RETREAT score of 0, and an additional one-third had a RETREAT score of 1. Thus, although overall RETREAT scores did correlate with post-transplant recurrence, the high number of patients with recurrence who had quite low RETREAT scores limited the sensitivity of the score for this purpose, and therefore limited the negative predictive value despite our overall relatively low rate of post-OLT recurrence. In our data, the negative predictive value was <95% using either a cutoff of a RETREAT score 0 or a cutoff of ≤1 to define the low-risk population. While a negative predictive value on the order of 95% seems appealing, a strategy of simply assuming no recurrence and offering no surveillance at all would carry a negative predictive value of 89% in our data, again due to the relatively low incidence of post-OLT recurrence regardless of the sensitivity and specificity of the RETREAT score.

Overall, in the absence of a clear evidence-based consensus among major medical society practice guidelines on the optimal post-OLT HCC surveillance strategy, and given our own center’s data on the limited sensitivity of the RETREAT score in predicting risk for post-OLT recurrence, as well as the lack of correlation between the RETREAT score and the time from transplant to HCC recurrence, our experience suggests applying a broad strategy of surveillance for all patients with HCC.

Furthermore, in the setting of post-OLT recurrence of HCC, important treatment considerations exist. Although combination immunotherapy regimens such as atezolizumab plus bevacizumab, durvalumab plus tremilimumab, and ipilimumab plus nivolumab based on the IM-Brave 150, HIMALAYA, and Checkmate 9DW trials, respectively, have yielded success in the general population of patients with advanced HCC, their utilization is typically avoided in the post-transplant setting due to graft rejection risk, with rates of rejection of up to 40% [24,25,26]. Given these concerns, anti-VEGF TKIs are typically recommended for frontline systemic treatment management after OLT recurrence. Although sorafenib had been the initial mainstay of systemic therapy after the pivotal SHARP trial, which demonstrated a significant improvement in overall survival (OS) versus best supportive care [27], this has been more commonly replaced with lenvatinib based on the findings of the REFLECT trial, which noted non-inferiority with sorafenib in terms of OS, but improved progression-free survival (PFS) and overall response rate (ORR), and potentially more manageable toxicities compared with sorafenib [28]. Our study also reflects this shift in treatment utilization patterns from sorafenib to lenvatinib over time. The safety profiles of both TKIs overlap, though lenvatinib has been associated with higher rates of hypertension and proteinuria, while sorafenib has been associated with a greater incidence of hand-foot syndrome and diarrhea [29]. Beyond sorafenib or lenvatinib, other TKI options include cabozantinib. The benefit of cabozantinib, which differs from other TKIs due to additional targets of MET and AXL, was demonstrated in the CELESTIAL study, where cabozantinib significantly improved overall survival compared with best supportive care (10.2 months vs. 8.0 months, respectively) in patients who received prior sorafenib, with some patients also receiving up to two prior lines of therapy [30]. Other agents with additional, though modest, benefit versus best supportive care post-sorafenib include regorafenib and ramucirumab [31,32]. Moreover, as observed in our patient cohort, attention needs to be paid to careful monitoring of the toxicities of these agents in the post-transplant setting.

Future directions in systemic therapy post-OLT recurrence may involve developing immunosuppression regimens that may balance the risk of graft rejection while permitting the safe use of checkpoint inhibitor therapy. Post-transplant immunotherapy has been investigated in the setting of other solid organ transplants. In a prospective trial by Hanna et al., twelve patients with advanced cutaneous squamous cell carcinoma after renal transplant were treated with a PD-inhibitor, cemiplimab, without graft rejection using a specific immunosuppression regimen utilizing mTOR inhibitors and dynamic prednisone dosing [33]. Most recently, a meta-analysis by Saleem et al. evaluated immunotherapy data post-transplant from 128 evaluable studies with a pooled number of 343 patients, of which the majority (70.9%) had renal transplants [34]. In this study, although there were patients who experienced acute graft rejection (36.2%) and graft loss (18.4%) at 1 year, there were also lower rejection rates when a combination of steroids and mTOR inhibitors was used for immunosuppression maintenance. Further prospective research is needed that may potentially balance the safety of checkpoint inhibitors with adequate immunosuppression post-OLT.

Checkpoint inhibitor immunotherapy has also emerged as a potential tool in the neoadjuvant setting to downstage tumors that are beyond the Milan Criteria for transplant. The prospective, multicenter VITALITy study examined patients being evaluated for liver transplant who received pre-OLT immunotherapy in addition to locoregional therapies [35]. In the VITALITy study, a 3-year survival rate of 71.1% was noted, as well as successful downstaging in 65 of 86 patients who initially were beyond the Milan Criteria. Of the 43 patients who ultimately underwent transplant, 7 experienced graft rejection; however, 6 of these patients had their last dose of ICI therapy within 3 months of transplant. An immunotherapy washout period of about 3 months has been proposed by AASLD in patients pre-transplant; however, routine utilization of immunotherapy pre-transplant has not been recommended for bridging purposes [7,36].

Our study does have several limitations, which include the dynamic nature of the HCC MELD exception policy, changes in liver transplant allocation policies, and median MELD scores over the time period of our study. We anticipate that such changes are likely to continue to evolve in the coming years, with substantive impact on access to cadaveric organs for transplant for patients with HCC, the relative utilization of living donor transplant for patients with HCC, an increase in the use of more marginal organs, and perhaps more utilization of therapies other than liver transplant due to access issues. Thus, rates of recurrence may change over time at our own center, raising questions about generalizability to other programs. An additional limitation regarding the generalizability of our findings is that different centers may have different approaches to patient selection for transplant in terms of attempting downstaging and offering transplants beyond the Milan Criteria. An additional limitation of our study is that we were unable to conduct a robust analysis of local disease control rates and complications relating to pre-transplant locoregional therapies due to the granularity of the data available in this retrospective study. We also acknowledge that the relatively small number of patients experiencing post-transplant recurrence of their HCC represents a limitation of our study. Given the relatively small number of recurrences, data regarding post-recurrence therapy is also limited to primarily descriptive analysis. The grading of symptom toxicities was challenging due to variability in clinical chart documentation resulting from the retrospective nature of this study.

## 5. Conclusions

Our single-center experience highlights the limitations in the sensitivity of the RETREAT score in identifying the risk of post-OLT HCC recurrence, with much of the negative predictive value of a low RETREAT score being driven by the overall fairly low rate of post-OLT recurrence. The utility of cost reduction by selecting patients at relatively low risk for post-OLT recurrence needs to be balanced against the benefits of early detection, taking into account unique challenges in managing post-OLT HCC recurrence, especially with respect to the provision of systemic therapy. Therefore, our center’s experience suggests caution in implementing a RETREAT score-based surveillance pathway for patients undergoing limited surveillance (or no surveillance) with low RETREAT scores.

## Figures and Tables

**Table 1:** Baseline characteristics of the study population, comparing those undergoing transplant with those without HCC.

		All	HCC	Non-HCC	*p*-Value
Number		923	329	594	
Age		58.6 mean 60.3 median	62.9 mean 63.5 median	56.3 mean 57.6 median	<0.01
Sex	Male	613 (66.4%)	264 (80%)	349 (59%)	<0.001
	Female	310 (33.6%)	65 (20%)	245 (41%)	
Race	White	715 (77.5%)	245 (75%)	470 (79%)	NS
	Black	111 (12.0%)	41 (13%)	70 (12%)	
	Hispanic	47 (5.1%)	21 (6%)	26 (4%)	
	Asian	37 (4.0%)	22 (7%)	15 (3%)	
	Other	13 (1.4%)	0 (0%)	13 (2%)	
Underlying Liver Disease	Viral	262 (28.4%)	195 (59%)	67 (11%)	<0.001
	Alcohol	264 (28.6%)	44 (13%)	220 (37%)	
	MASH	134 (14.5%)	49 (15%)	85 (14%)	
	Multiple/Other	263 (28.5%)	41 (13%)	222 (37%)	
Wait List Time		7.0 mean 4.5 median	8.6 mean 8.1 median	6.1 mean 1.6 median	<0.001
Clinical Follow-up Time		66.1 mean 60.4 median	78.5 mean 75.6 median	59.2 mean 53.3 median	<0.001

**Table 2:** Baseline characteristics of patients transplanted with HCC, comparing those experiencing post-transplant recurrence versus those not experiencing post-transplant recurrence.

		All	Recurrence	No Recurrence	*p* Value
Number		329 (100%)	36 (10.9%)	293 (89.1%)	
Age		62.9 mean 63.5 median	64.3 mean 66.1 median	62.7 mean 63.2 median	NS
Sex	Male	264 (80%)	31 (86.1%)	233 (79.5%)	NS
	Female	65 (20%)	5 (13.9%)	60 (20.5%)	
Race	White	245 (74.5%)	30 (83.3%)	215 (73.4%)	NS
	Black	41 (12.5%)	1 (2.8%)	40 (13.7%)	
	Hispanic	21 (6.4%)	3 (8.3%)	18 (6.1%)	
	Asian	19 (5.8%)	2 (5.6%)	20 (6.8%)	
	Other	0 (0%)	0 (0%)	0 (0%)	
Chronic Liver Disease	Viral	195 (59.3%)	19 (52.8%)	176 (60.1%)	NS
	Alcohol	44 (13.4%)	6 (16.7%)	38 (13.0%)	
	MASH	49 (14.9%)	6 (16.7%)	43 (14.7%)	
	Multiple/Other	41 (12.5%)	5 (13.9%)	36 (12.3%)	
Pre-OLT Treatment	Locoregional	291 (88.4%)	35 (97.2%)	256 (87.4%)	NS
	Downstaging	36 (10.9%)	6 (16.7%)	30 (10.2%)	NS
	Immunotherapy	0 (0%)	0 (0%)	0 (0%)	NS
Explant Findings	Any viable HCC	207 (62.9%)	31 (86.1)	176 (60.1%)	<0.002
	Viable HCC Beyond Milan	40 (12.2%)	13 (36.1%)	27 (9.2%)	<0.001
	Microvascular Invasion	17 (5.2%)	7 (19.4%)	10 (3.4%)	<0.001
	Diameter of largest lesion	1.1 cm mean 0.6 cm median Range 0–10 cm	2.5 cm mean 2.0 cm median Range 0–10 cm	0.9 cm mean 0.5 cm median Range 0–7 cm	<0.001
	Number of lesions	1.3 mean 1.0 median Range 1–20	2.0 mean 1 median Range 0–13	1.2 mean 1 median Range 0–20	NS
Highest degree of differentiation of HCC tumor on explant	No viable tumor	120 (36.5%)	5 (13.9%)	115 (39.2%)	<0.01
	Well differentiated	23 (7%)	3 (8.3%)	20 (6.8%)	
	Moderately differentiated	156 (47.4%	18 (50.0%)	138 (47.1%)	
	Poorly differentiated	9 (2.7%)	4 (11.1%)	5 (1.7%)	
	Unspecified/unknown	21 (6.4%)	6 (16.7%)	15 (5.1%)	
AFP		34.5 mean 5 median Range 1–2604	42.5 mean 8.5 median Range 1–290	33.4 mean 5 median Rance 1–2604	NS
RETREAT Score		1.1 mean 1.0 median range 0–6	2.4 mean 2.0 median Range 0–5	1.0 mean 1.1 median Range 0–6	<0.001

**Table 3:** Multivariate analysis of predictors of post-HCC recurrence.

Variable	Hazard Ratio	95% Confidence Interval	*p*-Value
RETREAT score (per point)	1.06	0.73–1.54	0.74
Presence of any viable HCC on explant (yes/no)	1.56	0.52–4.68	0.43
Presence of poorly differentiated HCC on explant (yes/no)	1.74	0.49–6.13	0.39
Presence of viable HCC beyond Milan Criteria on explant (yes/no)	2.25	0.93–5.49	0.07
Presence of microvascular invasion on explant (yes/no)	2.86	0.79–10.30	0.11
Diameter of largest focus of viable HC on explant (per cm)	1.19	0.97–1.47	0.09
Overall model: χ^2^ = 34.39 (df = 6), *p* < 0.001			

**Table 4:** Distribution of RETREAT scores in the population of patients undergoing OLT for HCC, comparing subgroups of patients with POST-OLT recurrence versus those without.

Score	All	Recurrence	No Recurrence	*p*-Value
0	117 (35.6%)	6 (16.7%)	111 (37.9%)	<0.001
1	121 (36.8%)	6 (16.7%)	115 (39.2%)	
2	49 (14.9%)	10 (27.8%)	39 (13.3%)	
3	22 (6.7%)	4 (11.1%)	18 (6.1%)	
4	7 (2.1%)	3 (8.3%)	4 (1.4%)	
5	11 (3.3%)	7 (19.4%)	4 (1.4%)	
6	2 (0.6%)	0 (0%)	2 (0.7%)	
7	0 (0%)	0 (0%)	0 (0%)	
8	0 (0%)	0 (0%)	0 (0%)	

**Table 5:** Performance of RETREAT score in predicting post-transplant recurrence, varying RETREAT score cutoffs defining higher versus lower risk. TP: true positive, FP: false positive, TN: true negative, FN: false negative, Sens: sensitivity, Spec: specificity, PPV: positive predictive value, NPV: negative predictive value, 95% CI: 95% confidence interval.

Low Risk	High Risk	TP	FP	TN	FN	Sens	Spec	PPV (95% CI)	NPV (95% CI)
0	1–8	30	182	111	6	83.33%	78.72%	14.15% (10.10–19.48%)	94.87% (89.26–97.63%)
0–1	2–8	24	67	226	12	66.67%	90.40%	26.37% (18.41–36.25%)	94.96% (91.40–97.09%)
0–2	3–8	14	28	265	22	38.89%	94.98%	33.33% (21.01–48.45%)	92.33% (88.67–94.88%)
0–3	4–8	10	10	283	26	27.78%	96.59%	50.00% (29.93–70.07%)	91.59% (87.96–94.19%)
0–4	5–8	7	6	287	29	19.44%	97.62%	53.85% (29.14–76.79%)	90.82% (87.13–93.53%)
0–5	6–8	0	2	291	36	0.00%	100%	0.00% (0–65.76%)	88.99% (85.14–91.94%)

**Table 6:** Overall post-transplant survival comparing groups of patients with versus without HCC.

	HCC	Non-HCC
1-year	97%	92%
3-year	88%	85%
5-year	80%	80%
10-year	69%	64%

**Table 7:** Overall post-transplant survival comparing groups of patients transplanted for HCC with versus without post-transplant HCC recurrence.

	With Post-OLT Recurrence	Without Post-OLT Recurrence
1-year	100%	97%
2-year	78%	94%
3-year	61%	92%

**Table 8:** Summary of sites of recurrence for the 36 patients experiencing post-transplant recurrence of HCC.

Initial Site of Identified HCC Recurrence		Number (%)
	Liver	8 (22.2)
	Lung	6 (16.7)
	Bone	4 (11.1)
	Nodal or Abdominal/Pelvic Mass	9 (25.0)
	Multiple	9 (25.0)
	Total	36 (100)

**Table 9:** Summary of treatments received for post-transplant HCC recurrence.

Treatments Received After Recurrence	Number
Locoregional Therapies	
TACE, TARE, MWA	12
External Beam Radiation	17
Surgery	5
Systemic Therapies (by treatment line):	
1st Line (total = 24)	
Lenvatinib	13
Sorafenib	10
Cytotoxic Chemotherapy	1
2nd Line (total = 13)	
Cabozantinib	7
Sorafenib	2
Regorafenib	1
Atezolizumab/Bevacizumab	1
Clinical Trial	2
3rd Line (total = 5)	
Cabozantinib	1
Lenvatinib	1
Cytotoxic Chemotherapy	3

## Data Availability

A copy of the full, de-identified data set is available upon request to the authors.

## Supplementary Materials

The following supporting information can be downloaded at https://www.mdpi.com/article/10.3390/cancers18050721/s1, Table S1: Subgroup analysis of patients restricted to a RETREAT score of 0 or 1, describing baseline characteristics of patients transplanted with HCC, comparing those experiencing post-transplant recurrence with those not experiencing post-transplant recurrence. Table S2: Subgroup analysis of patients undergoing transplant prior to 1 January 2020 (limiting subgroup to those patients having sufficient time to complete our center’s standard 5-year clinical protocol of surveillance for post-OLT recurrence): baseline characteristics of patients transplanted with HCC, comparing those experiencing post-transplant recurrence with those not experiencing post-transplant recurrence. Table S3: Subgroup analysis of patients undergoing transplant prior to 1 January 2020 (limiting subgroup to those patients having sufficient time to complete our center’s standard 5-year clinical protocol of surveillance for post-OLT recurrence): distribution of RETREAT scores in the population of patients undergoing OLT for HCC, comparing subgroups of patients with versus without POST-OLT recurrence. Table S4. Subgroup analysis of patients undergoing transplant prior to 1 January 2020 (limiting subgroup to those patients having sufficient time to complete our center’s standard 5-year clinical protocol of surveillance for post-OLT recurrence): multivariate analysis of predictors of post-HCC recurrence. Table S5. Patient therapies received for post-transplant HCC recurrence and immunosuppressive regimens.
