# Supplementary material for: Predicting and Managing Hepatocellular Carcinoma Recurrence After Liver Transplant: A Single-Center Experience 2012–2024
Source: Cancers (Basel). 2026 Feb 24;18(5):721. doi: 10.3390/cancers18050721 (PMC12984761; doi:10.3390/cancers18050721)
Supplement: Supplementary file 1 [file cancers-18-00721-s001.zip › cancers-4123961-supplementary.pdf]

cancers

MDPI

Supplemental Table S1. Subgroup analysis of patients restricted to a RETREAT score of 0 or 1, describing baseline characteristics of patients transplanted with HCC, comparing those experiencing post-transplant recurrence with those not experiencing post-transplant recurrence.

		All	Recurrence	No Recurrence	*p* Value
**Number**		238	12	226	
**Age (mean, years)**		62.8	63.7	62.7	NS
**Sex**	**Male**	188 (79%)	11 (91.7%)	177 (78.3%)	NS
	**Female**	50 (21%)	1 (8.3%)	49 (21.7%)	
**Race**	**White**	173 (72.7%)	10 (83.3%)	163 (72.1%)	NS
	**Black**	32 (13.4%)	1 (8.3%)	31 (13.7%)	
	**Hispanic**	16 (6.7%)	1 (8.3%)	15 (6.6%)	
	**Asian**	17 (7.1%)	0 (0%)	17 (7.5%)	
	**Other**	0 (0%)	0 (0%)	0 (0%)	
**Chronic Liver Disease**	**Viral**	146 (61.3%)	9 (75%)	137 (60.6%)	NS
	**Alcohol**	30 (12.6%)	0 (0%)	30 (13.3%)	
	**MASH**	37 (15.5%)	2 (16.7%)	35 (15.5%)	
	**Multiple/Other**	25 (10.5%)	1 (8.3%)	24 (10.6%)	
**Pre-OLT Treatment**	**Locoregional**	214 (89.9%)	12 (100%)	202 (89.4%)	NS
	**Downstaging**	25 (10.5%)	1 (8.3%)	24 (10.6%)	NS
	**Immunotherapy**	0 (0%)	0 (0%)	0 (0%)	NS
**Explant Findings**	**Any viable HCC**	120 (50.4%)	7 (58.3%)	113 (50.0%)	NS
	**Viable HCC Beyond Milan**	0 (0%)	0 (0%)	0 (0%)	NS
	**Microvascular Invasion**	1 (0.4%)	0 (0%)	1 (0.4%)	NS
	**Diameter of largest lesion**	0.60 cm mean<br>0 cm median	0.93 cm mean<br>0.11 cm median	0.56 cm mean<br>0 cm median	NS
	**Number of lesions**	0.64 mean<br>0 median	0.75 mean<br>1.0 median	0.61 mean<br>0 median	NS
**Highest degree of differentiation of HCC tumor on explant**	**No viable tumor**	117 (49.2%)	5 (41.7%)	112 (49.6%)	NS
	**Well differentiated**	19 (8%)	1 (8.3%)	18 (8.0%)	
	**Moderately differentiated**	88 (37%)	4 (33.3%)	84 (37.2%)	
	**Poorly differentiated**	0 (0%)	0 (0%)	0 (0%)	
	**Unspecified/unknown**	14 (5.9%)	2 (16.7%)	12 (5.3%)	
**AFP (mean)**		6.43 mean<br>5.0 median	6.33 mean<br>5.5 median	6.47 mean<br>5.0 median	NS

Supplemental Table S2. Subgroup analysis of patients undergoing transplant prior to 1 January 2020 (limiting subgroup to those patients having sufficient time to complete our center's standard 5-year clinical protocol of surveillance for post-OLT recurrence): baseline characteristics of patients transplanted with HCC, comparing those experiencing post-transplant recurrence with those not experiencing post-transplant recurrence.

		All	Recurrence	No Recurrence	*p* Value
**Number**		221	25	196	
**Age (years)**		62.1 mean<br>62.3 median	63.5 mean<br>65.1 median	61.7 mean<br>62.1 median	NS
**Sex**	**Male**	179 (81.0%)	21 (84.0%)	158 (80.6%)	NS
	**Female**	42 (19.0%)	4 (16.0%)	38 (19.4%)	
**Race**	**White**	168 (76.0%)	29 (80.0%)	148 (75.5%)	NS
	**Black**	24 (10.9%)	1 (4.0%)	23 (11.7%)	
	**Hispanic**	15 (6.8%)	3 (12.0%)	12 (6.1%)	
	**Asian**	14 (6.3%)	1 (4.0%)	13 (6.6%)	
	**Other**	0 (0%)	0 (0%)	0 (0%)	
**Chronic Liver Disease**	**Viral**	142(64.3%)	13 (52.0%)	129 (65.8%)	NS
	**Alcohol**	28 (12.7%)	4 (16.0%)	24 (12.2%)	
	**MASH**	25 (11.3%)	3 (12.0%)	22 (11.2%)	
	**Multiple/Other**	26 (11.8%)	5 (20%)	21 (10.7%)	
**Pre-OLT Treatment**	**Locoregional**	190 (86.0%)	24 (96.0%)	166 (84.7%)	NS
	**Downstaging**	22 (10.0%)	4 (16.0%)	18 (9.2%)	NS
	**Immunotherapy**	0 (0%)	0 (0%)	0 (0%)	NS
**Explant Findings**	**Any viable HCC**	147 (66.5%)	22 (88.0%)	125 (63.8%)	0.01
	**Viable HCC Beyond Milan**	30 (13.6%)	9 (36.0%)	21 (10.7%)	0.001
	**Microvascular Invasion**	11 (5.0%)	4 (16.0%)	7 (3.6%)	0.08
	**Diameter of largest lesion (cm)**	1.2 mean<br>0.8 median	2.8 mean<br>2.1 median	1.0 mean<br>0.6 median	0.001
	**Number of lesions**	1,2 mean<br>1.0 median	1.7 mean<br>1.0 median	1.2 mean<br>1.0 median	NS
**Highest degree of differentiation of HCC tumor on explant**	**No viable tumor**	72 (32.6%)	3 (12.0%)	69 (35.2%)	0.06
	**Well differentiated**	16 (7.2%)	2 (8.0%)	14 (7.1%)	
	**Moderately differentiated**	118 (53.4%)	17 (68.0%)	101 (51.5%)	
	**Poorly differentiated**	5 (2.3%)	2 (8.0%)	3 (1.5%)	
	**Unspecified/unknown**	10 (4.5%)	1 (4.0%)	9 (4.6%)	
**AFP**		43.2 mean<br>6.0 median	45.7 mean<br>8.0 median	42.8 mean<br>6.0 median	NS

Supplemental Table S3. Subgroup analysis of patients undergoing transplant prior to 1 January 2020 (limiting subgroup to those patients having sufficient time to complete our center's standard 5-year clinical protocol of surveillance for post-OLT recurrence): distribution of RETREAT scores in the population of patients undergoing OLT for HCC, comparing subgroups of patients with versus without POST-OLT recurrence.

Score	All	Recurrence	No Recurrence	*p*-Value
0	72 (32.6%)	3 (12%)	69 (35.2%)	< 0.01
1	85 (38.5%)	5 (20%)	80 (40.8%)	
2	19 (8.6%)	7 (28%)	26 (13.3%)	
3	19 (8.6%)	4 (16%)	15 (7.7%)	
4	4 (1.8%)	2 (8%)	2 (1.0%)	
5	6 (2.7%)	4 (16.0%)	2 (1.0%)	
6	2 (0.9%)	0 (0%)	2 (1.0%)	
7	0 (0%)	0 (0%)	0 (0%)	
8	0 (0%)	0 (0%)	0 (0%)	

Supplemental Table S4. Subgroup analysis of patients undergoing transplant prior to 1 January 2020 (limiting subgroup to those patients having sufficient time to complete our center's standard 5-year clinical protocol of surveillance for post-OLT recurrence): multivariate analysis of predictors of post-HCC recurrence.

Variable	Hazard Ratio	95% Confidence Interval	*p*-Value
RETREAT score (per point)	0.99	0.63–1.56	0.96
Presence of any viable HCC on explant (yes/no)	1.57	0.40–6.19	0.52
Presence of poorly differentiated HCC on explant (yes/no)	2.19	0.40–11.95	0.37
Presence of viable HCC beyond Milan Criteria on explant (yes/no)	1.66	0.55–5.00	0.37
Presence of microvascular invasion on explant (yes/no)	1.39	0.24–8.08	0.72
Diameter of largest focus of viable HC on explant (per cm)	1.36	1.05–1.76	0.02
Overall model: χ^2 = 22.06 (df = 6), $\boldsymbol{p}$ **= 0.001**			

Supplemental Table S5. Patient therapies received for post-transplant HCC recurrence and immunosuppressive regimens.

Patient	Immuno-Suppression	Received Treatment?	1L Systemic Treatment	Duration of 1L (mo)	Reason for Discontinuation 1L	2L Systemic Treatment	Duration of 2L (mo)	Reason for Discontinuation 2L	3L Systemic Treatment	Duration of 3L (mo)	Reason for Discontinuation 3L
1	tacrolimus	Locoregional + Systemic	Lenvatinib	5	Ongoing						
2	tacrolimus	None									
3	tacrolimus	Locoregional + Systemic	Lenvatinib	9	Progression	Atezolizumab/Bevacizumab	1	Toxicity			
4	tacrolimus	Locoregional + Systemic	Lenvatinib	3	Toxicity	Sorafenib	7	Progression	Cabozantinib	2	Toxicity
5	sirolimus	Locoregional + Systemic	Lenvatinib	4	Toxicity	Cabozantinib	2	Progression			
6	tacrolimus	None									
7	tacrolimus	None									
8	tacrolimus	Locoregional + Systemic	Lenvatinib	5	Progression	Cabozantinib	6	Death			
9	tacrolimus	Locoregional + Systemic	Sorafenib	5	Progression	Regorafenib	3	Toxicity			
10	tacrolimus	Locoregional									
11	sirolimus	Locoregional									
12	tacrolimus	Systemic	Lenvatinib	4	Progression	Cabozantinib	4	Progression	Gemcitabine/Oxaliplatin	1	Toxicity
13	unknown	Systemic	Sorafenib	4	Unknown						
14	sirolimus	Systemic	Lenvatinib	11	Toxicity						
15	tacrolimus + mycophenolate	Locoregional + Systemic	gem/ox	6	Progression	Sorafenib	3	Progression	Capecitabine	3	Progression
16	tacrolimus	Systemic	Lenvatinib	30	Progression	Cabozantinib	Unknown	Unknown			
17	sirolimus	Systemic	Sorafenib	8	Progression	Clinical Trial	Unknown	Toxicity	Lenvatinib	1	Progression
18	tacrolimus	Locoregional + Systemic	Lenvatinib	4	Progression	Cabozantinib	0.5	Toxicity			
19	tacrolimus	Locoregional									
20	tacrolimus	None									
21	sirolimus	Systemic	Sorafenib	4	Toxicity						
22	tacrolimus	Locoregional									

23	tacrolimus	Locoregional + Systemic	Sorafenib	4	Per Clinician						
24	tacrolimus	Locoregional + Systemic	Sorafenib	1	Toxicity						
25	tacrolimus	Locoregional + Systemic	Lenvatinib	7	Progression	Cabozantinib	1	Progression			
26	tacrolimus	Locoregional									
27	tacrolimus	Locoregional									
28	tacrolimus	Locoregional + Systemic	Lenvatinib	11	Toxicity						
29	tacrolimus	None									
30	tacrolimus	Locoregional + Systemic	Sorafenib	4	Progression	Clinical Trial	4	Progression	Gemcitabine/Oxaliplatin	8.5	Toxicity
31	sirolimus	Systemic	Sorafenib	3	Death						
32	tacrolimus	Systemic	Lenvatinib	0.5	Progression						
33	cyclosporine	Systemic	Sorafenib	Unknown	Unknown						
34	sirolimus	Systemic	Sorafenib	2	Progression						
35	tacrolimus	Locoregional + Systemic	Lenvatinib	4	Progression	Cabozantinib	Unknown	Toxicity			
36	tacrolimus	None									
